# Development of A Liquid Chromatography-Mass Spectrometry Technique for Evaluation of Multi-class Pesticides in Rice Samples

**DOI:** 10.22037/ijpr.2020.113071.14095

**Published:** 2021

**Authors:** Zohreh Mardani, Attaollah Shakoori, Farzad Peiravian, Leila Nouri, Jamshid Salamzadeh

**Affiliations:** a *Department of Food Science and Technology, Damghan Branch, Islamic Azad University, Damghan, Iran. *; b *Vice-Chancellor for Food and Drugs Affairs, Shahid Beheshti University of Medical Sciences, Tehran, Iran. *; c *Department of Pharmacoeconomics and Pharma Management, School of Pharmacy, Shahid Beheshti University of Medical Sciences, Tehran, Iran. *; d *Food Safety Research Center, Shahid Beheshti University of Medical Sciences, Tehran, Iran.*

**Keywords:** Pesticide, Residues, LC-MS/MS, Rice, Iran

## Abstract

In the current study, a liquid chromatography coupled mass detector was set up to detect and quantify 108 pesticide residues in rice samples. QuEChERS method was applied for sample preparation and different validation parameters were determined to ensure the suitability of the method. The calibration curves were linear in the concentration 0.01-1.00 mg/kg with a coefficient of determination (R^2^) of more than 0.990 for all compounds. Based on signal to noise studies, the calculated LODs and LOQs were 0.005-0.060 mg/kg and 0.018-0.199 mg/kg, respectively; and acquired mean recoveries at three spiked levels (0.025, 0.200 and 0.800 mg/kg) were 72% - 117% with RSD < 20%. The developed method was used to investigate the occurrence of the studied pesticides in 65 internal and 65 foreign rice samples. The results showed that 14 internal and 15 imported samples were found to be contaminated 12 pesticides in the amounts between 0.027 mg/kg to 0.078 mg/kg and 0.031 mg/kg to 0.081 mg/kg, respectively. According to the Iranian regulations, with the exception of nine prohibited pesticides for rice production in Iran, bioallethrin, cypermethrin, deltamethrin, flutriafol, foramsulfuron, imazalil, phosphamidon, TCMTB, and triasulfuron, three permitted pesticides, cinosulfuron, triadimenol, and tricyclazole, found in positive rice samples were below MRLs established by Iranian National Standard Organization (INSO).

## Introduction

In recent decades, the use of pesticides has dramatically increased worldwide. The combined effects of pesticides with several means such as the production of new cereal varieties, increasing cultivated land, increased fertilizer use, and irrigation have increased world food production to double (1).

Pesticides prevent animal and plant diseases and remarkably reduce the devastation of crops, and are used to get better the yield of harvests such as rice and vegetables (2). However, they also cause public concern due to their potential adverse effects on human health. Pesticides cause acute and chronic toxicity. In 2017, the United Nations reported that pesticide acute exposures annually resulted in 200,000 deaths in the world, and 99% death cases occurred in developing countries, due to weaker safety, health and environmental regulations (3). Previously, a similar investigation had shown that acute pesticide exposures were responsible for 3 million hospitalized cases with nearly 220,000 deaths in the world (4). Nonetheless, chronic exposures to pesticides has been remained one of the main challenges in the human societies and may include carcinogenesis in adults (5) and children (6, 7), teratogenesis (8), liver disorders (9), endocrine disruption (10), and reproductive toxicity (11).

Pesticides are not completely destroyed in environment and their residues can pollute soil, water and crops, and ﬁnally the contaminated foodstuffs are consumed by different groups of society (12). Pesticides are used in various stages of crop production, and most residues appear in foodstuffs due to the direct application of a pesticide to a crop or the treatments of food materials. Pesticide residues not only occur in crops but also in animal products such as meat, milk and eggs because of the ingestion of contaminated feed by farm animals. Human exposure to pesticides can occur through dermal contact, oral route, and inhalation. Among them, the oral route is the most common way (13). Despite the toxic effects of pesticides, the use of them for protection and control of crops against pests is inevitable. Therefore, the use of pesticides requires proper management, especially; their oral intake should be restricted. For these reasons, different limiting parameters for pesticides in foodstuffs like maximum residue limits (MRL) have been set up in various countries such as Iran (14) and the European Union (15-17). 

Analysis of pesticide residues in foods is a great challenge, because they usually present at low levels in complex matrices (18). Therefore, the control and management of pesticide residues in foods require powerful analytical methods. In recent decades, various advanced instruments have been made for tracing pesticides at nanogram per gram levels in different foods. Among them, gas and liquid chromatography techniques coupled to different detectors especially mass spectrometer (MS), have been widely used in the world. Gas chromatography (GC) methods are suitable for volatile and thermally stable chemicals. Hence, analysis of thermally unstable or non-volatile pesticides; for instance, benzimidazoles and carbamates, using GC systems is very complicated or inconceivable. To overcome the mentioned challenge, liquid chromatography (LC) combine with diverse detectors chiefly tandem mass spectrometer (MS-MS) provides a powerful tool for analysis of these chemicals in food matrices (19). Despite less selectivity and sensitivity of traditional UV detectors and DADs, in recent years, different ionization techniques *e.g.* atmospheric pressure ionization (API), along with MS instruments have been invented. Taken together, these techniques not only increase the sensitivity of the analysis but also provide cheaper and simpler clean-up procedures (20).

Rice is a popular and main food in the world; including Iran, and every year millions tonnes of this valuable crop are produced. In recent decades, due to the increasing importance of food security, rice production and consumption has been grown in the world and Iran. The application of various pesticides during different stages of cultivation is one of the reasons for increasing rice production (21). Therefore, pesticide residues in rice are predictable, and long-term consumption of contaminated rice can affect human health. For the above reasons, continuous monitoring of pesticides in rice absolutely is an essential need, and obviously, this monitoring requires advanced analytical procedures.

The objects of this investigation were firstly the development of a multi-residue method for the determination of 108 multi-class pesticides in rice, using LC-MS/MS, secondly applying of the validated method for detection and determination of the studied pesticide residues in domestic and imported rice samples collected from Tehran market, IR Iran.

## Experimental

*Chemicals*

All standards of the studied pesticides (Table S1, Supplementary file); triphenylphosphate (TPP) and anhydrous magnesium sulfate (MgSO4) were obtained from Sigma–Aldrich (Germany). Carbofuran-d3 (C-D3) as a surrogate was purchased from Dr. Ehrenstorfer (Germany) and Ammonium formate, methanol (MeOH), and acetonitrile (MeCN) from Acros (Belgium). Ethyl acetate (EtAc), acetic acid (HOAc), and sodium acetate were supplied from Merck (Darmstadt, Germany). Bondesil-primary secondary amine (PSA, 40 μm) was provided from Interchim (France). HPLC grade water (H_2_O) was freshly provided by Milli-Q Plus ultra-pure water system (Millipore, Molsheim, France).

*Preparations of standards*

According to the solubility of the studied pesticides at 20 ◦C, their standard stock solutions (1000 μg/mL) were prepared in EtAc, with the exception of carbendazim in dimethylformamide, chlormequat, and mepiquat in MeCN and Cartap and Fuberidazole in MeOH. A mixed standard solution (5 μg/mL) was prepared via appropriate dilution of the stock solutions in MeOH containing 0.1% HOAc. A 20 μg/mL solution of triphenylphosphate (TPP) and carbofuran-d3 (C-D3) in EtAc, were applied as internal standard and surrogate, respectively, and an aliquot of 50 μL of their solutions (20 μg/mL) was added to the spiked rice sample. The investigated pesticides were selected according to the Iranian National Standard Organization (INSO) regulations, NO.13120 in cereals or commonly detected pesticides in accordance with the international reports, like of EU and FDA during recent years (14).

*Rice sample collection*

One hundred and thirty rice samples, including 65 domestic and 65 imported, were collected from different regions of Tehran, in April-May 2019. A mix of 100 g of the rice samples and 100 g of dry ice were ground, right after purchase and stored in a fridge at -20 °C until analysis.

*Sample preparation*

Sample preparation procedures were carried out by the original QuEChERS method (22). Five grams of homogenized rice sample was weighed into a 50 mL centrifuge tube followed by the addition of internal standard (TPP), C-D3 (0.1 mg/kg) as a surrogate, and for spiking purposes, appropriate amounts of the mixed working standard solution. Then, 10 mL of MeCN was added and vigorously shacked for 2 min. After the addition of 4 g MgSO4 and 1.5 g sodium acetate, the contents were intensively agitated for 2.0 min again. The mixture was centrifuged for 5 min at 5433×g, and 5 mL of the supernatants were evaporated in a nitrogen evaporator at 40 °C until dryness. The residue was reconstructed in 0.5 mL MeCN and vortex mixed for 2.0 min, followed by sonication for 4.0 min. The solution transferred to a micro-tube containing 60 mg anhydrous MgSO_4 _and 20 mg PSA, then vortex mixed vigorously for 2 min and centrifuged for 5 min at 5433×g. Finally, an appropriate amount of the cleaned extract was transferred into a vial, and 100 μL of the solution was injected into LC-MS/MS.

*Liquid chromatography*

The analysis of the different pesticides from the samples was accomplished using an Alliance separations module 2695 (Waters, Milford, USA), which consist of a quaternary solvent delivery system, degasser, autosampler, column heater, and diode array detector coupled with a Quattro Micro Triple Quadrupole mass spectrometer (Waters, Micromass, Manchester, UK). Chromatographic separation was performed using an Agilent ZORBAX Eclipse XDB-C_18_ (Narrow-Bore 2.1 × 150 mm, 3.5-micron) analytical column at a ﬂow rate of 1.0 mL/min and an injection volume of 100 μL. The mobile phase was 5 mM ammonium formate in methanol (solvent A) and 5 mM ammonium formate in water (solvent B) in a gradient mode and a total analysis time of 30 min. The elution program was as follows: at the start, 30% solvent A and 70% solvent B; the percentage of solvent A was linearly increased to 100% in 20 min, then remained constant for 5 min and ramped to original composition in 5 min. The column temperature was kept constant at 40 °C.

*Mass spectrometry*

 The triple quadrupole mass detector contained an electrospray source (Z-spray) and analysis was performed in positive ionization mode. MassLynx software, version 4.0, was applied for data acquisition. The electrospray ionization (ESI) parameters were: capillary voltage, 4.12 kV; extractor, 2 V; RF lens, 0.1 V; source temperature, 120 ◦C; desolvation temperature, 300 ◦C; desolvation gas and cone gas (nitrogen 99.99% purity) ﬂow rates, 600 and 50 L/h, respectively. The analyzer parameters were: resolution, 14.6 (unit resolution) for LM1 and LM2 resolution and 14 for HM1 and HM2 resolution; ion energy 1 and 2, 0.3 and 3.0, respectively; entrance and exit energies, 55 and 75 (V); multiplier, 700 (V); collision gas (argon, 99.995%) pressure 5.35 × 10^-3 ^mbar. MS/MS conditions for all pesticides were directed in the positive ionization mode applying multiple reaction monitoring (MRM) with two mass transitions. The product ion with the strongest intensity was used for quantitation, while the other with the lowest intensity was employed for conﬁrmation.

*Method validation*

The method was validated to assess for linearity, matrix effects, limit of quantiﬁcation (LOQ), the limit of detection (LOD), accuracy, and precision. Linearity was studied applying spiked calibration curve by analyzing in triplicate six concentration levels, between 0.01 and 1.00 mg/kg. For evaluating the matrix effect, six different concentrations of standards were analyzed in solvent and in the matrix, and the slopes of the calibration curves were compared. Mean recoveries and precisions were calculated by using five spiked blank samples at three concentration levels of 0.025, 0.200, and 0.800 mg/kg on three different days. The LOQs and LODs were estimated based on the signal-to-noise ratio (S/N). The amounts of the studied pesticides in rice samples were obtained by interpolation of the relative peak areas for each pesticide to the internal standard peak area (herein, TTP) in the same sample on the spiked calibration curve. Surrogate (C-D_3_) was used in addition to the internal standard (TPP) in order to better control the assay at all stages of sample preparation and instrumental analysis.

## Results

 The linearity of the method was assessed using spiked calibration curves at six levels over the range of 0.01 to 1.00 mg/kg. As shown in Table S2, the linearity within the studied range was very good for all the studied chemicals, with coefficient of determinations (R^2^) higher than 0.990 in all cases (81.5% ≥ 0.995).

Accuracy and repeatability (precision) of the method were calculated by recoveries and RSD percentage. As shown in Table S3, the mean recoveries obtained for all pesticides at three spike levels were in the range of 72-117%, and RSDs were in the range of 1-18%.

According to the signal-to-noise studies, the estimated LODs and LOQs for analyzed pesticides were 0.005-0.060 mg/kg and 0.018-0.199 mg/kg, respectively (Table S3).

In this study for evaluating matrix effects, the obtained slopes in case of spiked calibration curve and solvent-based calibration curve were compared and matrix effects were calculated by means of the following Equation (26):

Matrix effect (%) = (1 - Slope (matrix)/slope (solvent)) × 100

**Figure 1:**
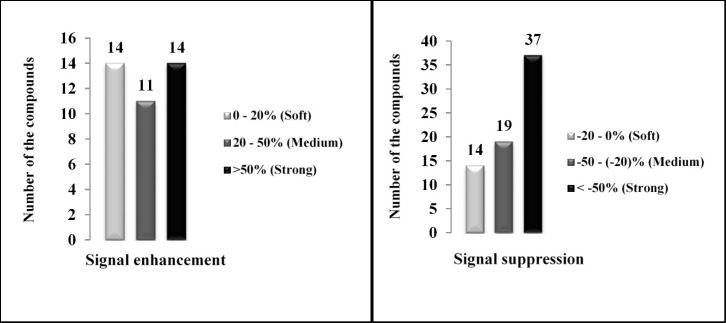
Distribution of matrix effects in rice samples

As illustrated in Figure 1, all of the investigated pesticides presented matrix effect where ion suppression and enhancement occurred in 70 compounds (64%) and 39 compounds (36%), respectively.

The validated method was applied for the analysis of 130 rice samples, including 65 domestic and 65 imported, collected from different local markets of 22 different regions in Tehran. The results indicated that 14 internal and 15 imported samples were found to be contaminated 12 pesticides in the amounts between 0.027 mg/kg to 0.078 mg/kg and 0.031 mg/kg to 0.081 mg/kg, respectively.

## Discussion

*LC- MS/MS determination*

The initial liquid chromatography (LC) method was set up using a methanol and water composition. This mobile phase composition gave very poor response for most pesticides. Therefore, a combination of 0.5 mM ammonium formate/MeOH and H2O containing 0.1% formic acid was used. In the acidic condition, some pesticides gave a better response but it was noticed that formation of sodium adducts as a new challenge, suppressed some pesticide responses such as acephate, acetochlor and alachlor. On the other hand, some pesticides, such as cypermethrin gave no response. Some previous studies have shown that the formation of the sodium adducts is suppressed by using ammonium formate buffer (23, 24). Therefore, the gradient profile of 5-mM ammonium formate in MeOH and 5-mM ammonium formate in H2O was used as the final mobile phase. In this situation, most of the pesticides formed [M+H]+ precursor ions, pesticides belonging to quarternary ammonium group including chlormequat and mepiquat appeared as [M]+, and cypermethrin formed ammonium adduct parent ions.

Optimization of MS parameters has a key role in multi-residue analysis and the successful performance of a developed method strongly depends on this stage. The MS parameters of the studied pesticides individually were optimized to obtain the parent ion molecules (precursor ions) and selecting two transitions (daughter ions) with higher molecular mass and better intensity for avoiding the interference of the matrix components. All of the pesticides were optimized in the positive ESI mode and multiple reactions monitoring (MRM). Experiments were directed with a dwell time and inter-channel delay of 0.06 s and 0.1 s, respectively. The optimization of the parent ions, daughter ions, cone voltage (CV) and collision energy (CE) was done through direct injection of the single pesticide standard solutions (1 μg/mL in ammonium formate 5 mM/MeOH) into the mass spectrometer applying a syringe pump at flow rate 10 μL/min. After optimization, CVs and CEs of different transitions were in the range 10-53 (V) and 8-45 (eV), respectively. For each chemical, two transitions or daughter ions were selected, the most intense transition for quantitation, and the other for conﬁrmation (Table S1). The time-scheduled data acquisition sequence was conducted in eight functions, ranged from 22 to 32 MRM channels.

In order to achieve maximum responses (parent and daughter ions), different parameters of the MS ion source such as capillary voltage (4.12 V), source temperature (120^◦^C), desolvation gas temperature (300 ◦C) were optimized. Desolvation gas and cone gas (nitrogen) ﬂow-rates were set at 50 L/h and 600 L/h, respectively. Pesticides were identified in accordance with their retention times, target (1^st^ transition), qualifier (2^nd^ transition) ions and ion ratios (the ratio of the intensity of 1st transition to 2^nd^ transition). The quantitation was based on the peak area ratio of the targets to that of the internal standard. Table S1 gives a summary of the mass data obtained for the studied pesticides in MRM mode.

*Matrix effects*

Co-extraction of matrix components with desired analytes (matrix effects) is a major challenge in food residue analysis. Matrix effects result to signal suppression or enhancement of target compounds and can strongly influence quantitative analysis of chemicals at low levels; also, it can extremely affect the performance of the method (25). Agricultural products, including rice, have a complex structure due to their various molecules. Therefore, in the analysis process, rice molecules can interfere with analyte molecules and falsely increase or decrease the signal intensity of the analytes (false positive or false negative). Matrix effects were estimated applying the ratio of the slopes of spiked calibration curves and solvent-based calibration curves.

 If the percentage of calculated matrix effect is positive, signal enhancement occurs and if it is negative, it indicates signal suppression. Depending on the obtained values of the matrix effects formula, various matrix effects could be observed. In this study, the resulted matrix effects were classified into soft, moderate and strong. Soft matrix effect occurred when an evaluated percentage was between -20% and 0% or between 0% and 20%. When the values were between -50% and -20% or 20% and 50%, medium matrix effects occurred. The percentage values less than -50% or above 50% considered as a strong matrix effect (Table S2).

The results showed that all the studied compounds presented matrix effect in the form of ion enhancement or suppression (Figure 1). In the case of suppression, 14 out of 108 compounds (13%) presented soft matrix effect while medium and strong matrix effects observed for 19 compounds (17%) and 37 compounds (34%), respectively. Soft, medium and strong signal enhancement occurred in 14 (13%), 11 (10%) and 14 (13%) studied pesticides, respectively. Thus, for overcoming matrix effect and avoiding any under or over estimations, spiked calibration curves were plotted for determination of studied pesticides.

*Validation studies*

**Figure 2:**
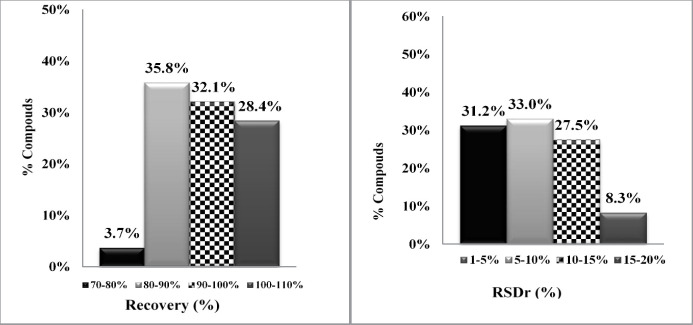
Total recoveries (Left) and RSDs obtained (Right) for investigated pesticides at three spike levels

The regression equation (y = ax + b) and coefficient of determinations (R^2^) was applied to assessed the linearity. The analytical calibration curves showed that R^2^ values were greater than 0.990. Therefore, there was a very good linear relationship between the concentrations of the studied chemicals and the areas under their chromatograms. Spiked rice samples at three levels *i.e.* 0.025, 0.200 and 0.80 mg/kg were prepared for evaluating of the pesticide recoveries. Extraction method was performed five times at each spiking level in three different days (each day 15, totally 45 spike levels). For data analysis, the ratio of a pesticide peak area/ TPP peak area was calculated and the concentration of each pesticide determined by spiked calibration curves. In each series of analysis, a blank rice sample was used. As shown in Table S3, mean and total recoveries and RSDs at three spiked levels were calculated and results indicated that 96% recoveries were in the range of 80-110% and 92% and RSDs were below 15% (Figure 2). The recoveries and repeatabilities, for all pesticide, were in the acceptable range recommended by the SANTE/12682/2019 the European Quality Control Guidelines *i.e.*70–120% for recoveries and RSD < 20% (27).

*Surrogate spike control of the method*

Stability of a validated method may gradually change in routine residue analyses. The changes may occur at all stages of the analysis and in most cases are completely hidden. If these likely alterations are not recognized, they cause serious errors in the obtained results. As a practical approach, surrogate spikes are used to prove an assay method is in control. These chemicals are distinctly different to the analytes but are very similar in chemical properties and have the same manners during sample preparation and analysis (28). Among chemicals, deuterated analogs of the analytes can be ideal surrogates because chemically, they behave like the analytes and cannot be present in the sample originally. These compounds are added at a known concentration to the samples and the blanks prior to sample preparation and supply a measure of the complete efficiency of the method. Practically, the surrogate recoveries are calculated for any run and unusually high or low recoveries indicate a difficulty, such as pollution or instrument fault (29).

**Figure 3:**
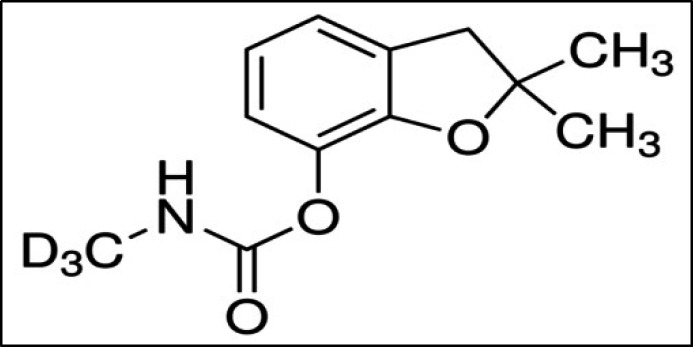
Chemical structure of carbofuran-d3

In the present study, C-D_3_ was used as surrogate (Figure 3). This compound was spiked to the blanks, the real samples and the quality control samples at concentration of 0.200 mg/kg. According to Table S3, total mean recovery obtained for C-D_3 _was 96% with RSD of 2%. 

*Application of the method to real samples*

One of the aims of this study was to use the developed method for the analysis of the investigated pesticide residues in real rice samples collected from different local markets of Tehran. Iran is one of the most important rice producing countries in the world. However, due to high domestic consumption, it also imports large quantities of rice annually. Therefore, both domestic and imported rice samples (65 samples from each group) were collected and analyzed based on the developed method. In order to prevent the molecular degradation of pesticides, the rice samples were immediately transferred to the laboratory and stored in the freezer (-20 ◦C) until analysis.

The results indicated that 29 of domestic or imported rice samples (22%) were contaminated with 12 pesticides. As shown in Table S4, in domestic samples bioallethrin was the most common pesticide residue detected (found in 7.7% of samples) followed by deltamethrin, triadimenol, cinosulfuron, cypermethrin, foramsulfuron, imazalil tricyclazole. In imported rice samples, phosphamidon was the most common pesticide residue detected (6.1% of samples) followed by triasulfuron, bioallethrin, TCMTB, cinosulfuron, flutriafol triadimenol and tricyclazole.

**Figure 4:**
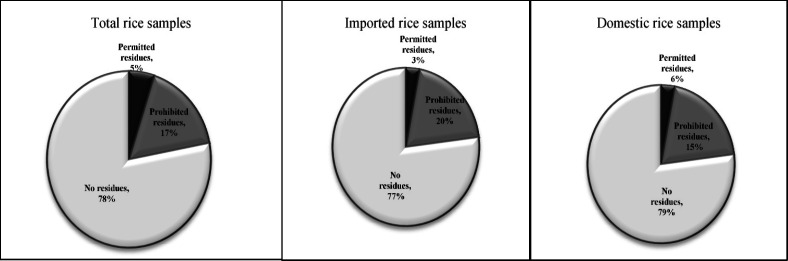
The percentage of contamination to permitted and prohibited pesticides in total, domestic and imported rice samples

From 12 detected pesticides in rice samples, according to Iranian regulation, three pesticides including cinosulfuron, triadimenol and tricyclazole are permitted to be used for rice production. In this study, the concentrations of these pesticides were below MRLs. However, the nine other detected pesticides are prohibited for rice cultivation in Iran and their presence in the samples is great concern. In domestic samples, permitted and prohibited pesticides were found in 6% and 15% of the samples, respectively. On the other hand, in imported samples, 3% and 20% of the samples were polluted with permitted and prohibited pesticides, respectively. In total, from 130 samples, permitted and prohibited pesticides were found in 5% and 17% of samples, respectively (Figure 4).

## Conclusion

A multi-residue LC-MS/MS method using QuEChERS sample preparation was developed and applied for simultaneous analysis of 108 pesticides in rice samples. Validation studies showed excellent recoveries and repeatabilities with good linearity for all the chemicals. Matrix effect studies showed signal suppressions or enhancements for all analytes. Thus, the use of spiked calibration curves reduced adverse matrix-related effects. The developed method was used for analyses of 130 real rice samples. Twelve pesticides were detected and determined in positive samples. Seven samples were contaminated with cinosulfuron, triadimenol and/or tricyclazole at the levels below Iranian maximum residue limits (MRLs) in rice. Twenty two out of one hundred thirty (17%) samples were contaminated with prohibited pesticides.

## Supplementary Materials

Supplement
