# Supplementary material for: Development of A Liquid Chromatography-Mass Spectrometry Technique for Evaluation of Multi-class Pesticides in Rice Samples
Source: Iran J Pharm Res. 2021 Winter;20(1):165–74. doi: 10.22037/ijpr.2020.113071.14095 (PMC8170775; doi:10.22037/ijpr.2020.113071.14095)
Supplement: Supplement [file ijpr-20-165-s001.pdf]

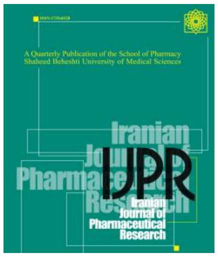

Supplementary Materials for

Development of A Liquid Chromatography-Mass Spectrometry Technique for Evaluation of Multi-class Pesticides in Rice Samples

Zohreh Mardani[a, b], Attaollah Shakoori[b], Farzad Peiravian[c], Leila Nouri[a*] and Jamshid Salamzadeh[d*]

*To whom correspondence should be addressed. E-mail: nouri.le.ir@gmail.com; j.salamzadeh@sbmu.ac.ir

Volume 20, Issue 1 (Winter 2021)

This PDF file includes:
Tables S1-S4

Table S1. Names, molar masses, chromatographic and MRM parameters for studied pesticides.

No.	Compound	MW (g/mol)	Rt (min)	Parent ion	CV (V)	1st Transition (Quantitation)	CE (eV)	2nd Transition (Confirmation)	CE (eV)	Ion ratio
1	Acephate	183	2.95	$[M+H]^+$	15	184 ⟶143	8	184⟶125	30	16.4
2	Acetochlor	269	19.93	$[M+H]^+$	20	270⟶224	15	270⟶148	15	1.5
3	Alachlor	269	19.95	$[M+H]^+$	22	270⟶238	17	270⟶162	17	1.5
4	Atrazine	215	15.72	$[M+H]^+$	36	216⟶174	20	216⟶96	24	1.1
5	Azinphos-ethyl [b]	345	18.99	$[M+H]^+$	15	346⟶77	36	346⟶132	30	1.4
6	Azinphos-methyl	317	17.99	$[M+H]^+$	20	318⟶160	10	318⟶261	10	11.3
7	Azoxystrobin	403	18.31	$[M+H]^+$	22	404⟶372	15	404⟶329	30	2.5
8	Benalaxyl	325	21.57	$[M+H]^+$	30	326⟶91	20	326⟶316	34	1.2
9	Bioallethrin	302	24.13	$[M+H]^+$	20	303⟶123	15	303⟶151	10	1.7
10	Bitertanol	337	21.32	$[M+H]^+$	20	338⟶70	17	338⟶99	8	4.7
11	Bromacil [b]	260	13.75	$[M+H]^+$	20	261⟶205	12	261⟶188	35	7.0
12	Buprofezin	305	24.34	$[M+H]^+$	30	306⟶57	20	306⟶201	15	2.8
13	Carbaryl [a]	201	14.61	$[M+H]^+$	15	202⟶145	20	202⟶117	10	3.9
14	Carbendazim	191	9.48	$[M+H]^+$	30	192⟶160	20	192⟶132	26	3.1
15	Carbofuran [b]	221	15.00	$[M+H]^+$	15	222⟶165	16	222⟶123	16	1.4
16	Carbofuran-d3[*]	224	14.17	$[M+H]^+$	15	225⟶123	30	225⟶165	10	1.4
17	Carbosulfan	380	26.87	$[M+H]^+$	40	381⟶118	25	381⟶76	34	1.6
18	Carboxin	235	14.61	$[M+H]^+$	32	236⟶143	16	236⟶87	22	3.4
19	Cartap [a]	237	2.57	$[M+H]^+$	27	238⟶73	16	238⟶150	16	1.8
20	Chlorbromuron [b]	292	18.47	$[M+H]^+$	28	293⟶204	16	293⟶182	16	1.3
21	Chlorfenvinphos [b]	358	21.05	$[M+H]^+$	28	359⟶99	28	359⟶155	17	2.1
22	Chlormequat	122	2.66	$[M]^+$	20	122 ⟶58	20	124 ⟶58	20	3.1
23	Chlorpyrifos[a]	350	25.24	$[M+H]^+$	30	350⟶97	25	350⟶198	22	2.1
24	Chlorpyrifos-methyl	321	22.93	$[M+H]^+$	34	322⟶125	20	322⟶290	18	2.7
25	Cinosulfuron[a]	413	5.67	$[M+H]^+$	16	414⟶183	15	414⟶157	15	17.9
26	Clodinafob-propargyl	349	20.18	$[M+H]^+$	20	350⟶266	22	350⟶91	20	1.4
27	Clofentezine	302	22.04	$[M+H]^+$	25	303⟶102	35	303⟶138	33	1.3
28	Coumaphos[b]	362	20.95	$[M+H]^+$	35	363⟶307	17	363⟶289	25	3.2
29	Cycloxydim	325	17.59	$[M+H]^+$	28	326⟶280	20	326⟶180	15	1.3
30	Cypermethrin	415	25.70	$[M+NH4]^+$	15	433⟶191	15	433⟶127	41	5.0
31	Cyproconazole	291	20.29	$[M+H]^+$	30	292⟶125	28	292⟶70	20	2.7
32	Cyprodinil	225	22.04	$[M+H]^+$	40	226⟶93	38	226⟶108	27	1.3
33	Deltamethrin	505	25.73	$[M+H]^+$	30	506⟶281	44	506⟶93	12	3.0
34	Dialifos[b]	393	22.77	$[M+H]^+$	20	394⟶187	10	394⟶208	15	1.1
35	Diazinon[a]	304	21.92	$[M+H]^+$	29	305⟶97	35	305⟶169	20	1.9
36	Dicrotophos[b]	237	4.32	$[M+H]^+$	26	238⟶112	10	238⟶193	10	3.3
37	Difenoconazole	405	22.02	$[M+H]^+$	40	406⟶251	25	406⟶337	20	16.7
38	Dimethenamid	275	17.61	$[M+H]^+$	23	276⟶244	18	276⟶168	24	2.5
39	Dimoxystrobin	326	19.98	$[M+H]^+$	25	327⟶116	12	327⟶205	20	2.6

40	Diniconazole	325	22.50	$[M+H]^+$	15	326⟶70	30	326⟶159	34	15.6
41	Disulfoton	274	22.79	$[M+H]^+$	18	275⟶89	22	275⟶61	35	2.4
42	Edifenphos[a]	310	20.50	$[M+H]^+$	30	311⟶109	32	311⟶111	26	4.9
43	Ethion	384	24.39	$[M+H]^+$	20	385⟶97	10	385⟶199	43	1.2
44	Ethoprophos	242	19.49	$[M+H]^+$	26	243⟶97	28	243⟶131	15	1.2
45	Etrimfos	292	21.67	$[M+H]^+$	35	293⟶125	25	293⟶265	18	2.7
46	Fenamiphos	303	19.77	$[M+H]^+$	32	304⟶217	25	304⟶202	32	2.0
47	Fenarimol	330	19.78	$[M+H]^+$	20	331⟶268	20	331⟶81	20	2.6
48	Fenbuconazole	336	19.58	$[M+H]^+$	32	337⟶70	16	337⟶125	35	1.6
49	Fenhexamid	301	19.76	$[M+H]^+$	41	302⟶97	22	302⟶55	31	1.8
50	Fenoxaprop-P-ethyl	361	23.64	$[M+H]^+$	20	362⟶121	25	362⟶288	25	1.2
51	Fenpropathrin	349	25.14	$[M+H]^+$	20	350⟶125	15	350⟶97	20	4.2
52	Fenpyroximate	421	25.59	$[M+H]^+$	32	422⟶366	15	422⟶138	35	1.9
53	Flamprop-M-Isopropyl	363	20.43	$[M+H]^+$	20	364⟶105	20	364⟶77	50	2.7
54	Fluometuron[b]	232	15.33	$[M+H]^+$	30	233⟶72	18	233⟶46	20	4.9
55	Fluoxastrobin	458	19.56	$[M+H]^+$	30	459⟶188	36	459⟶427	24	1.3
56	Flutriafol	301	16.35	$[M+H]^+$	32	302⟶70	25	302⟶123	20	1.8
57	Foramsulfuron	452	6.05	$[M+H]^+$	32	453⟶182	20	453⟶272	15	4.5
58	Fuberidazole	184	12.21	$[M+H]^+$	42	185⟶157	25	185⟶156	32	2.2
59	Haloxyfop	361	23.50	$[M+H]^+$	10	362⟶91	35	362⟶316	12	46.3
60	Hexythiazox	352	24.85	$[M+H]^+$	26	353⟶228	15	353⟶168	30	2.0
61	Imazalil	296	20.39	$[M+H]^+$	35	297⟶69	30	297⟶159	22	1.1
62	Imazamethabenz-methyl	288	14.13	$[M+H]^+$	10	289⟶144	26	289⟶161	26	1.5
63	Iprobenfos[b]	288	20.19	$[M+H]^+$	20	289⟶91	18	289⟶205	14	6.7
64	Isoproturon	206	16.62	$[M+H]^+$	35	207⟶72	18	207⟶47	18	69.5
65	Linuron	248	18.20	$[M+H]^+$	25	249⟶160	15	249⟶181	15	13.6
66	Malathion[a]	330	18.43	$[M+H]^+$	18	331⟶127	12	331⟶99	20	1.4
67	Mepiquat	114	2.73	$[M]^+$	30	114 ⟶58	25	114 ⟶70	25	4.6
68	Mesosulfuron-methyl	503	8.64	$[M+H]^+$	38	504⟶182	23	504⟶83	60	2.0
69	Metalaxyl	279	16.79	$[M+H]^+$	24	280⟶192	17	280⟶220	20	3.0
70	Methabenzthiazuron[b]	221	15.53	$[M+H]^+$	28	222⟶165	20	222⟶150	30	2.2
71	Methidathion[b]	302	16.93	$[M+H]^+$	18	303⟶145	20	303⟶85	10	1.2
72	Monocrotophos[b]	223	3.73	$[M+H]^+$	26	224⟶127	18	224⟶98	14	2.3
73	Omethoate[b]	213	3.10	$[M+H]^+$	20	214⟶125	18	214⟶183	15	4.4
74	Oxadiazon[a]	344	24.27	$[M+H]^+$	30	345⟶220	13	345⟶177	40	1.5
75	Oxydemeton-methyl[a]	246	3.26	$[M+H]^+$	20	247 ⟶109	25	247 ⟶169	14	1.3
76	Penconazole	283	20.71	$[M+H]^+$	28	284⟶70	20	284⟶159	20	1.7
77	Phosalone	367	22.32	$[M+H]^+$	20	368⟶182	38	368⟶111	20	1.2
78	Phosmet	317	17.68	$[M+H]^+$	30	318⟶160	30	318⟶77	43	3.1
79	Phosphamidon[b]	299	12.47	$[M+H]^+$	26	300⟶127	20	300⟶174	10	2.7
80	Phoxim	298	21.46	$[M+H]^+$	16	299⟶129	13	299⟶153	11	5.9
81	Pinoxaden	400	21.15	$[M+H]^+$	10	401⟶317	17	401⟶57	17	2.3
82	Prallethrin	300	23.06	$[M+H]^+$	15	301⟶105	8	301⟶133	8	1.6

83	Primicarb	238	15.34	$[M+H]^+$	27	239⟶72	25	239⟶182	15	4.2
84	Primiphos-methyl[a]	305	22.44	$[M+H]^+$	30	306⟶108	28	306⟶164	17	4.8
85	Prochloraz	375	22.17	$[M+H]^+$	20	376⟶308	15	376⟶266	20	6.1
86	Profenofos	372	23.96	$[M+H]^+$	36	373⟶303	42	373⟶128	20	2.2
87	Propiconazole[a]	341	21.26	$[M+H]^+$	40	342⟶159	30	342⟶69	16	1.0
88	Propoxur[b]	209	13.93	$[M+H]^+$	20	210⟶111	14	210⟶168	8	3.2
89	Pyraclostrobin	387	21.31	$[M+H]^+$	26	388⟶194	14	388⟶163	22	1.1
90	Pyrazophos[b]	373	22.13	$[M+H]^+$	36	374⟶222	30	374⟶194	21	1.3
91	Spinosyn A[a]	732	26.65	$[M+H]^+$	53	733⟶142	30	733⟶98	56	3.7
92	Spinosyn D[a]	746	27.44	$[M+H]^+$	50	747⟶142	31	747⟶98	51	4.4
93	Spiroxamine	297	22.86	$[M+H]^+$	40	298⟶144	20	298⟶100	35	1.3
94	Sulfosulfuron	470	8.02	$[M+H]^+$	25	471⟶211	13	471⟶261	18	1.4
95	TCMTB[b]	238	18.10	$[M+H]^+$	21	239⟶180	10	239⟶136	40	3.6
96	Tebuconazole	307	20.80	$[M+H]^+$	35	308⟶70	20	308⟶125	45	16.9
97	Thiobencarb[a]	257	22.57	$[M+H]^+$	18	258⟶125	20	258⟶100	13	7.7
98	Thiodicarb[a]	354	15.38	$[M+H]^+$	20	355⟶88	16	355⟶108	13	1.5
99	Thiophanate-methyl[a]	342	13.51	$[M+H]^+$	24	343⟶151	22	343⟶311	12	6.1
100	Triadimefon	293	19.41	$[M+H]^+$	30	294⟶69	20	294⟶197	15	2.9
101	Triadimenol[a]	295	19.66	$[M+H]^+$	20	296⟶70	8	296⟶99	15	7.4
102	Triallate	303	25.34	$[M+H]^+$	32	304⟶86	20	304⟶143	24	1.1
103	Triasulfuron	401	8.59	$[M+H]^+$	30	402⟶141	20	402⟶167	17	1.0
104	Triazophos[b]	313	18.85	$[M+H]^+$	31	314⟶162	18	314⟶119	32	1.8
105	Tricyclazole[a]	189	10.40	$[M+H]^+$	38	190⟶136	27	190⟶163	22	1.4
106	Trifloxystrobin	408	22.40	$[M+H]^+$	30	409⟶186	16	409⟶206	16	3.3
107	Triflumizole[a]	345	23.17	$[M+H]^+$	10	346⟶278	8	346⟶73	25	2.4
108	Triflumuron	358	21.20	$[M+H]^+$	30	359⟶156	35	359⟶139	21	1.2
109	Triphenylphosphate[**]	326	20.81	$[M+H]^+$	20	327⟶152	45	327⟶77	45	1.9
110	Triticonazole	317	18.46	$[M+H]^+$	15	318⟶70	14	318⟶125	28	2.2

[*]Surrogate.

[**]Internal standard.

[a]Permitted pesticides in Iran for rice production.

[b]Prohibited pesticides in Iran.

Table S2. Linearity and matrix effects of studied compounds.

No.	Pesticides	Solvent-base calibration curve		Matrix-matched calibration curve		A*	Matrix Effect (%)**
		Slope	R^2	Slope	R^2		
1	Acephate	0.415	0.992	0.717	0.996	1.728	-72.77
2	Acetochlor	0.111	0.997	0.097	0.997	0.874	12.61
3	Alachlor	0.118	0.997	0.134	0.992	1.136	-13.56
4	Atrazine	0.781	0.997	0.761	0.999	0.974	2.56
5	Azinphos-ethyl	0.466	0.999	0.789	0.996	1.693	-69.31
6	Azinphos-methyl	1.212	0.998	1.305	0.998	1.077	-7.67
7	Azoxystrobin	1.612	0.998	1.608	0.995	0.998	0.25
8	Benalaxyl	0.678	0.997	1.124	0.994	1.658	-65.78
9	Bioallethrin	1.274	0.999	0.365	0.995	0.286	71.35
10	Bitertanol	0.890	0.997	1.610	0.999	1.809	-80.90
11	Bromacil	1.001	0.996	0.256	0.994	0.256	74.43
12	Buprofezin	1.160	0.998	2.051	0.990	1.768	-76.81
13	Carbaryl	2.891	0.996	0.945	0.998	0.327	67.31
14	Carbendazim	1.150	0.999	2.030	0.998	1.765	-76.52
15	Carbofuran	1.875	0.999	2.459	0.996	1.311	-31.15
16	Carbofuran-d3	1.995	0.998	2.220	0.995	1.113	-11.28
17	Carbosulfan	0.868	0.996	1.462	0.993	1.684	-68.43
18	Carboxin	1.424	0.997	1.065	0.997	0.748	25.21
19	Cartap	1.104	0.993	0.050	0.998	0.045	95.47
20	Chlorbromuron	0.281	0.996	0.520	0.994	1.851	-85.05
21	Chlorfenvinphos	1.564	0.995	2.273	0.991	1.453	-45.33
22	Chlormequat	0.329	0.993	0.212	0.993	0.644	35.56
23	Chlorpyrifos	1.869	0.996	2.363	0.999	1.264	-26.43
24	Chlorpyrifos-methyl	0.714	0.998	0.907	0.998	1.270	-27.03
25	Cinosulfuron	0.293	0.993	0.061	0.998	0.208	79.18
26	Clodinafob-propargyl	0.344	0.999	0.346	0.995	1.006	-0.58
27	Clofentezine	0.404	0.994	0.587	0.997	1.453	-45.30
28	Coumaphos	0.535	1.000	0.755	0.999	1.411	-41.12
29	Cycloxydim	0.137	0.996	0.031	0.996	0.226	77.37
30	Cypermethrin	0.216	0.991	0.146	0.995	0.676	32.41
31	Cyproconazole	3.962	0.998	4.711	0.998	1.189	-18.90
32	Cyprodinil	2.041	0.993	1.990	0.997	0.975	2.50
33	Deltamethrin	0.594	0.993	0.424	0.998	0.714	28.62
34	Dialifos	0.718	0.997	0.825	0.997	1.149	-14.90
35	Diazinon	3.167	0.997	5.019	0.999	1.585	-58.48
36	Dicrotophos	2.380	0.993	1.294	0.999	0.544	45.63
37	Difenoconazole	2.559	0.998	3.798	0.993	1.484	-48.42
38	Dimethenamid	1.559	0.998	2.026	0.999	1.300	-29.96
39	Dimoxystrobin	2.346	0.999	3.950	0.998	1.684	-68.37
40	Diniconazole	0.586	0.999	0.889	0.999	1.517	-51.71

41	Disulfoton	0.290	0.995	0.385	1.000	1.328	-32.76
42	Edifenphos	3.127	0.997	5.548	0.999	1.774	-77.42
43	Ethion	3.057	0.999	5.307	0.999	1.736	-73.60
44	Ethoprophos	1.757	0.996	1.823	0.998	1.038	-3.76
45	Etrimfos	3.056	0.999	4.789	0.999	1.567	-56.71
46	Fenamiphos	2.857	0.999	4.577	0.996	1.602	-60.20
47	Fenarimol	0.186	1.000	0.260	0.998	1.398	-39.78
48	Fenbuconazole	1.923	0.997	2.653	0.999	1.380	-37.96
49	Fenhexamid	1.506	0.998	0.383	0.996	0.254	74.57
50	Fenoxaprop-P-ethyl	3.800	0.991	2.368	0.999	0.623	37.68
51	Fenpropathrin	0.988	0.999	1.883	0.996	1.906	-90.59
52	Fenpyroximate	5.685	0.997	4.509	0.999	0.793	20.69
53	Flamprop-M-Isopropyl	2.285	0.994	3.254	0.994	1.424	-42.41
54	Fluometuron	1.793	0.998	1.654	0.998	0.922	7.75
55	Fluoxastrobin	0.370	0.998	0.455	0.997	1.230	-22.97
56	Flutriafol	0.999	0.993	1.054	0.996	1.055	-5.51
57	Foramsulfuron	0.396	0.995	0.006	0.994	0.015	98.48
58	Fuberidazole	1.646	0.998	1.086	0.999	0.660	34.02
59	Haloxyfop	0.425	0.998	0.692	0.996	1.628	-62.82
60	Hexythiazox	2.684	0.997	1.961	0.999	0.731	26.94
61	Imazalil	2.950	0.994	2.877	0.994	0.975	2.47
62	Imazamethabenz-methyl	0.215	0.992	0.183	0.990	0.851	14.88
63	Iprobenfos	4.296	0.999	6.598	0.998	1.536	-53.58
64	Isoproturon	2.252	0.995	2.182	0.996	0.969	3.11
65	Linuron	0.434	0.995	0.626	0.996	1.442	-44.24
66	Malathion	2.355	0.998	4.078	0.999	1.732	-73.16
67	Mepiquat	0.660	0.995	0.715	0.991	1.083	-8.33
68	Mesosulfufuron-methyl	0.519	0.997	0.099	0.998	0.191	80.92
69	Metalaxyl	0.458	0.998	0.457	0.993	0.998	0.22
70	Methabenzthiazuron	1.377	0.997	1.627	0.998	1.182	-18.16
71	Methidathion	1.202	0.998	1.537	0.997	1.279	-27.87
72	Monocrotophos	0.834	0.993	1.331	0.996	1.596	-59.59
73	Omethoate	0.718	0.995	1.261	0.998	1.756	-75.63
74	Oxadiazon	0.719	0.994	0.768	0.997	1.068	-6.82
75	Oxydemeton-methyl	1.073	0.999	1.717	0.995	1.600	-60.02
76	Penconazole	1.107	0.994	1.821	0.998	1.645	-64.50
77	Phosalone	1.036	0.993	0.881	0.991	0.850	14.96
78	Phosmet	0.245	0.996	0.217	0.996	0.886	11.43
79	Phosphamidon	1.591	0.995	2.660	0.999	1.672	-67.19
80	Phoxim	1.087	0.996	1.300	0.998	1.196	-19.60
81	Pinoxaden	0.293	0.999	0.131	0.993	0.447	55.29
82	Prallethrin	0.517	0.996	0.842	0.999	1.629	-62.86
83	Primicarb	3.945	0.998	7.353	0.999	1.864	-86.39

84	Primiphos-methyl	9.040	0.999	11.646	0.998	1.288	-28.83
85	Prochloraz	0.752	1.000	1.456	0.997	1.936	-93.62
86	Profenofos	1.048	0.998	1.936	0.997	1.847	-84.73
87	Propiconazole	0.737	0.998	1.290	0.998	1.750	-75.03
88	Propoxur	1.296	0.998	1.353	0.998	1.044	-4.40
89	Pyraclostrobin	0.645	1.000	0.986	0.994	1.529	-52.87
90	Pyrazophos	1.350	0.996	1.263	0.998	0.936	6.44
91	Spinosyn A	5.935	0.995	2.129	0.999	0.359	64.13
92	Spinosyn D	2.568	0.999	2.141	0.997	0.834	16.63
93	Spiroxamine	14.060	0.997	6.319	0.997	0.449	55.06
94	Sulfosulfuron	0.183	0.996	0.249	0.999	1.361	-36.07
95	TCMTB	0.782	0.995	1.091	0.999	1.395	-39.51
96	Tebuconazole	3.933	0.998	6.499	0.996	1.652	-65.24
97	Thiobencarb	2.241	0.998	2.686	0.998	1.199	-19.86
98	Thiodicarb	1.126	0.996	1.079	0.994	0.958	4.17
99	Thiophanate-methyl	0.915	0.996	0.245	0.994	0.268	73.22
100	Triadimefon	1.290	0.996	2.230	0.998	1.729	-72.87
101	Triadimenol	0.564	0.997	0.953	0.993	1.690	-68.97
102	Triallate	1.137	0.998	0.873	0.998	0.768	23.22
103	Triasulfuron	0.152	0.997	0.047	0.998	0.309	69.08
104	Triazophos	2.902	1.000	5.211	0.998	1.796	-79.57
105	Tricyclazole	0.972	1.000	1.689	0.998	1.738	-73.77
106	Trifloxystrobin	1.407	0.996	2.321	0.997	1.650	-64.96
107	Triflumizole	0.928	0.991	1.429	0.999	1.540	-53.99
108	Triflumuron	0.386	0.999	0.525	0.995	1.360	-36.01
109	Triticonazole	2.118	0.999	1.542	0.999	0.728	27.20

*A = Slope (matrix)/slope (solvent).

**Matrix Effect (%) = (1 - A) × 100.

Table S3. Mean recoveries (%), RSDs[a] (%), LODs[b] and LOQs[c] of studied pesticides in rice samples, spiked at 0.025, 0.200 and 0.800 mg/kg levels (n = 5).

NO.	Compound	0.025 (mg/kg)		0.200 (mg/kg)		0.800 (mg/kg)		LOD (mg/kg)	LOQ (mg/kg)
		Mean	RSD	Mean	RSD	Mean	RSD		
1	Acephate	84	15	78	8	85	13	0.010	0.032
2	Acetochlor	113	9	86	17	79	5	0.013	0.043
3	Alachlor	105	18	83	10	77	8	0.008	0.028
4	Atrazine	82	11	89	17	84	5	0.008	0.025
5	Azinphos-ethyl	90	4	85	8	81	2	0.012	0.038
6	Azinphos-methyl	89	6	84	7	80	3	0.007	0.024
7	Azoxystrobin	98	9	101	10	103	9	0.010	0.032
8	Benalaxyl	102	18	98	10	94	11	0.012	0.038
9	Bioallethrin	102	8	92	10	90	11	0.013	0.043
10	Bitertanol	96	18	96	15	98	14	0.011	0.037
11	Bromacil	104	5	79	5	81	8	0.008	0.028
12	Buprofezin	93	1	111	7	94	3	0.010	0.033
13	Carbaryl	96	5	84	8	81	3	0.011	0.037
14	Carbendazim	92	5	84	13	86	13	0.013	0.043
15	Carbofuran	81	8	82	7	84	9	0.013	0.042
16	Carbofuran-d3	95	8	94	10	98	6	0.011	0.036
17	Carbosulfan	109	5	108	2	104	2	0.007	0.023
18	Carboxin	72	4	91	14	83	13	0.007	0.022
19	Cartap	92	18	101	16	99	9	0.008	0.027
20	Chlorbromuron	88	15	84	7	95	6	0.012	0.039
21	Chlorfenvinphos	83	2	96	11	94	9	0.009	0.031
22	Chlormequat	100	4	102	6	105	3	0.007	0.022
23	Chlorpyrifos	111	2	81	7	82	10	0.008	0.025
24	Chlorpyrifos-methyl	109	4	89	10	98	4	0.009	0.029
25	Cinosulfuron	86	7	91	18	91	7	0.011	0.036
26	Clodinafob-propargyl	96	8	91	18	88	4	0.007	0.022
27	Clofentezine	111	6	112	2	95	11	0.009	0.030
28	Coumaphos	98	10	105	11	87	5	0.011	0.035
29	Cycloxydim	108	5	100	6	102	1	0.010	0.033
30	Cypermethrin	89	8	83	3	86	6	0.008	0.026
31	Cyproconazole	101	5	86	8	90	10	0.009	0.029
32	Cyprodinil	115	1	96	1	97	11	0.006	0.019
33	Deltamethrin	85	10	81	3	83	3	0.007	0.023
34	Dialifos	116	3	103	9	87	9	0.006	0.021
35	Diazinon	109	1	98	4	101	11	0.007	0.023
36	Dicrotophos	94	13	92	11	98	8	0.008	0.025
37	Difenoconazole	112	3	108	7	105	8	0.012	0.038
38	Dimethenamid	111	3	100	4	89	11	0.010	0.033
39	Dimoxystrobin	94	7	85	10	84	9	0.012	0.038
40	Diniconazole	92	2	93	5	91	5	0.007	0.022

41	Disulfoton	112	2	96	10	99	18	0.012	0.041
42	Edifenphos	72	3	95	10	89	8	0.014	0.045
43	Ethion	90	3	106	8	109	6	0.010	0.032
44	Ethoprophos	102	11	103	10	109	6	0.010	0.034
45	Etrimfos	103	3	92	8	96	7	0.008	0.027
46	Fenamiphos	115	5	94	4	92	11	0.008	0.026
47	Fenarimol	109	2	88	4	84	4	0.004	0.014
48	Fenbuconazole	117	6	102	3	109	2	0.009	0.029
49	Fenhexamid	110	1	95	6	100	3	0.007	0.024
50	Fenoxaprop-P-ethyl	78	8	74	5	77	7	0.008	0.026
51	Fenpropathrin	117	1	107	5	100	14	0.012	0.038
52	Fenpyroximate	90	4	83	8	79	11	0.013	0.044
53	Flamprop-M-Isopropyl	77	4	86	14	87	11	0.011	0.037
54	Fluometuron	77	3	94	10	97	6	0.005	0.018
55	Fluoxastrobin	78	7	93	16	92	9	0.008	0.028
56	Flutriafol	72	3	83	8	97	12	0.004	0.014
57	Foramsulfuron	112	4	98	7	95	5	0.006	0.021
58	Fuberidazole	109	6	85	12	79	9	0.011	0.036
59	Haloxyfop	84	3	103	5	100	2	0.006	0.021
60	Hexythiazox	102	8	84	14	79	5	0.008	0.028
61	Imazalil	110	2	83	9	103	7	0.006	0.019
62	Imazamethabenz-methyl	90	5	91	9	81	5	0.005	0.017
63	Iprobenfos	92	5	95	10	96	10	0.007	0.024
64	Isoproturon	86	4	87	9	89	10	0.008	0.027
65	Linuron	82	5	89	13	87	8	0.011	0.036
66	Malathion	90	4	90	16	94	9	0.009	0.031
67	Mepiquat	106	3	107	3	110	2	0.005	0.016
68	Mesosulfufuron-methyl	77	3	89	7	90	5	0.005	0.015
69	Metalaxyl	75	5	83	7	75	7	0.008	0.028
70	Methabenzthiazuron	99	8	81	6	77	6	0.005	0.016
71	Methidathion	110	5	98	10	93	7	0.010	0.032
72	Monocrotophos	79	16	101	5	103	6	0.013	0.044
73	Omethoate	100	14	93	12	98	10	0.008	0.027
74	Oxadiazon	100	3	100	6	94	10	0.011	0.036
75	Oxydemeton-methyl	88	18	110	7	115	5	0.010	0.034
76	Penconazole	94	2	89	5	93	5	0.010	0.032
77	Phosalone	95	2	110	7	111	5	0.008	0.025
78	Phosmet	78	4	81	15	80	11	0.006	0.020
79	Phosphamidon	103	6	77	8	87	10	0.009	0.029
80	Phoxim	78	12	102	14	100	6	0.010	0.033
81	Pinoxaden	82	15	79	6	77	8	0.006	0.019
82	Prallethrin	111	6	100	6	87	6	0.010	0.034
83	Primicarb	79	2	86	9	84	7	0.012	0.038

84	Primiphos-methyl	117	3	96	3	101	8	0.005	0.015
85	Prochloraz	116	2	104	6	110	1	0.006	0.021
86	Profenofos	109	2	101	5	106	6	0.008	0.025
87	Propiconazole	107	3	87	6	89	12	0.010	0.034
88	Propoxur	75	7	86	18	83	12	0.009	0.031
89	Pyraclostrobin	107	3	103	14	99	15	0.010	0.033
90	Pyrazophos	108	2	92	9	90	3	0.011	0.037
91	Spinosyn A	102	9	94	17	100	18	0.009	0.029
92	Spinosyn D	84	13	92	12	95	7	0.006	0.020
93	Spiroxamine	114	6	100	16	91	9	0.007	0.022
94	Sulfosulfuron	74	3	82	8	89	6	0.010	0.032
95	TCMTB	104	4	81	13	84	13	0.012	0.040
96	Tebuconazole	96	1	96	4	107	7	0.013	0.043
97	Thiobencarb	104	4	100	10	95	8	0.012	0.040
98	Thiodicarb	100	9	108	7	107	17	0.011	0.035
99	Thiophanate-methyl	81	6	110	12	92	14	0.010	0.034
100	Triadimefon	80	5	88	16	96	11	0.008	0.028
101	Triadimenol	78	1	76	8	94	7	0.012	0.038
102	Triallate	75	5	80	14	78	5	0.005	0.018
103	Triasulfuron	82	12	83	6	94	5	0.008	0.026
104	Triazophos	98	9	81	15	81	9	0.008	0.027
105	Tricyclazole	89	4	82	15	79	10	0.010	0.033
106	Trifloxystrobin	116	3	100	18	92	18	0.010	0.034
107	Triflumizole	79	12	84	13	95	12	0.011	0.036
108	Triflumuron	78	5	108	14	97	14	0.010	0.034
109	Triticonazole	105	5	84	11	92	9	0.007	0.024

[a]Relative Standard Deviations.

[b]Limit of Quantitation.

[c]Limit of Detection.

Table S4. Numbers and concentration ranges of pesticides found in domestic and imported rice samples analyzed with INSO's MRL (mg/kg).

No.	Pesticides	No. of positive samples		Range (mg/kg)		Mean[c] (mg/kg)		MRL (mg/kg)
		Domestic	Imported	Domestic	Imported	Domestic	Imported	
1	Bioallethrin[b]	5 (7.7%)	2 (3.1%)	0.030-0.078	0.042-0.068	0.017	0.016	-------
2	Cinosulfuron[a]	1 (1.5%)	1 (1.5%)	0.034	0.047	0.010	0.011	0.05
3	Cypermethrin[b]	1 (1.5%)	-------	0.010	-------	0.010	-------	-------
4	Deltamethrin[b]	2 (3.1%)	-------	0.048-0.061	-------	0.011	-------	-------
5	Flutriafol[b]	-------	1 (1.5%)	-------	0.038	-------	0.010	-------
6	Foramsulfuron[b]	1 (1.5%)	-------	0.043	-------	0.043	-------	-------
7	Imazalil[b]	1 (1.5%)	-------	0.035	-------	0.035	-------	-------
8	Phosphamidon[b]	------	4 (6.1%)	-------	0.044-0.081	-------	0.012	-------
9	TCMTB[b]	-------	2 (3.1%)	-------	0.031-0.050	-------	0.010	-------
10	Triadimenol[a]	2 (3.1%)	1 (1.5%)	0.027-0.046	0.044	0.010	0.044	0.5
11	Tricyclazole[a]	1 (1.5%)	1 (1.5%)	0.038	0.034	0.038	0.034	5.0
12	Triasulfuron[b]	-------	3 (4.6%)	-------	0.051-0.073	-------	0.011	-------

[a]Permitted pesticides in Iran for rice production.

[b]Prohibited pesticides in Iran for rice production.

[c]Mean of total samples.
